# Usefulness of thalamic beta activity for closed-loop therapy in essential tremor

**DOI:** 10.1038/s41598-023-49511-5

**Published:** 2023-12-15

**Authors:** Dominique Guehl, Etienne Guillaud, Nicolas Langbour, Emilie Doat, Nicolas Auzou, Edouard Courtin, Olivier Branchard, Julien Engelhardt, Abdelhamid Benazzouz, Alexandre Eusebio, Emmanuel Cuny, Pierre Burbaud

**Affiliations:** 1 Service de Neurophysiologie Clinique de l’enfant et de l’adulte, Hôpital Pellegrin, Pôle des Neurosciences Cliniques, CHU de Bordeaux, Bordeaux, France; 2 Institut des Maladies Neurodégénératives, Univ. Bordeaux, CNRS, IMN, UMR 5293, F-33000, Bordeaux, France; 3 Centre de Recherche en Psychiatrie, CH de la Milétrie, 86000 Poitiers, France; 4 Institute of Cognitive and Integrative Neurosciences, Univ. Bordeaux, CNRS, INCIA, UMR 5287, F-33000, Bordeaux, France; 5 Institut des Maladies Neurodégénératives Clinique (IMNc), Pôle des Neurosciences Cliniques, CHU de Bordeaux, Bordeaux, France; 6 Service de Neurochirurgie, CHU de Bordeaux, Bordeaux, France; 7 Department of Neurology and Movement Disorders, APHM, Hôpitaux Universitaire de Marseille, Marseille, France; 8 Institut de Neurosciences de la Timone, UMR 7289, Aix Marseille Univ, CNRS, Marseille, France

**Keywords:** Neurology, Movement disorders

## Abstract

A partial loss of effectiveness of deep brain stimulation of the ventral intermediate nucleus of the thalamus (VIM) has been reported in some patients with essential tremor (ET), possibly due to habituation to permanent stimulation. This study focused on the evolution of VIM local-field potentials (LFPs) data over time to assess the long-term feasibility of closed-loop therapy based on thalamic activity. We performed recordings of thalamic LFPs in 10 patients with severe ET using the ACTIVA™ PC + S (Medtronic plc.) allowing both recordings and stimulation in the same region. Particular attention was paid to describing the evolution of LFPs over time from 3 to 24 months after surgery when the stimulation was Off. We demonstrated a significant decrease in high-beta LFPs amplitude during movements inducing tremor in comparison to the rest condition 3 months after surgery (1.91 ± 0.89 at rest vs. 1.27 ± 1.37 µV^2^/Hz during posture/action for N = 8/10 patients; p = 0.010), 12 months after surgery (2.92 ± 1.75 at rest vs. 2.12 ± 1.78 µV^2^/Hz during posture/action for N = 7/10 patients; p = 0.014) and 24 months after surgery (2.32 ± 0.35 at rest vs 0.75 ± 0.78 µV^2^/Hz during posture/action for 4/6 patients; p = 0.017). Among the patients who exhibited a significant decrease of high-beta LFP amplitude when stimulation was Off, this phenomenon was observed at least twice during the follow-up. Although the extent of this decrease in high-beta LFPs amplitude during movements inducing tremor may vary over time, this thalamic biomarker of movement could potentially be usable for closed-loop therapy in the long term.

## Introduction

Essential tremor (ET) is the most frequent abnormal movement encountered in the general population with a prevalence of 0.9%^1^. Its pathophysiology remains a matter of debate, but a line of evidence suggests a disruption of the cerebello-thalamo-cortical pathway encompassing the dentate nucleus, the ventral intermediate nucleus of the thalamus (VIM) and the primary motor cortex^2^. Several hypotheses have been evoked to account for tremor induction such as a dysfunction of the inferior olive, degeneration, or heterotopia of Purkinje cells (CP), swelling and reduction in their dendrites, remodeling of cerebellar basket cells and CP axons (torpedos), or rearrangement of climbing fibers^3^. In addition, a decrease in the glutamate transporter type EAAT2A produced by astrocytes^4^ and a reduction of GABA receptors in the dentate nucleus^5^ have also been reported. Thanks to imaging studies, two hypotheses have emerged^6^: the existence of an abnormal cerebellar oscillator; and a decoupling between the cerebellum and the motor cortex.

Pharmacological treatment of severe ET remains disappointing, which explains the development of alternative therapeutic strategies such as thalamotomy^7^ or deep brain stimulation (DBS) targeting the VIM. The latter is the relay of cerebellar projections to the cerebral cortex. This approach provides good improvement of ET with 33 to 82% of patients responding to stimulation^8,9^. However, some patients report a partial loss of the benefit of VIM-DBS over time. The explanation for this phenomenon remains unclear but it could be due to disease progression or habituation to chronic stimulation^10^.

To date, DBS has been based on continuous high frequency stimulation, which does not take subjects’ motor status into account. An alternative would be to propose intermittent stimulation triggered by internal or external events, a strategy which relies on a more physiological approach. This approach has been tested with success in Parkinson’s disease (PD) using subthalamic nucleus (STN) local field potential (LFP) in the beta band or cortical narrowband gamma (60–90 Hz) oscillation as biomarkers of the disease^11–13^. Beta burst rates have also been described as a potential biomarker in the sensorimotor cortex (SMC) and STN of PD patients^14^. In ET, attempts so far have concerned external markers of tremor amplitude and phase measured by electromyographic electrodes or accelerometers positioned on the arm^15–17^. However, in these cases, VIM-DBS was triggered by tremor and did not precede its onset. While having an internal trigger would avoid recourse to external devices, there would be a need for a reliable intracerebral biomarker of ET that is stable over time^18^. Recently, different teams recorded thalamic LFP in ET and reported neuronal oscillations in the alpha and theta frequency bands using either temporary externalized leads following surgery or micro/macroelectrodes during the surgical procedure^19–22^. Over a period of 6 months after surgery, Opri et al. tested closed-loop stimulation for VIM DBS (CL-DBS) in three patients with ET. They delivered VIM stimulation based on upper limb motor activity detected with a cortical electrode and demonstrated that it was as effective as open-loop stimulation^23^.

In the present study, our goal was to characterize intra-thalamic oscillations independently of the frequency band in patients with ET and to determine whether these oscillations persist over time, with a view to using them as potential biomarkers for closed-loop therapy in the future. Thus, we chronically recorded LFP in the thalamus of 10 patients operated for severe ET and followed a part of them up to 24 months. We used an implantable device (ACTIVA™ PC + S, Medtronic plc.) allowing both chronic deep brain stimulation and LFP recordings in natural conditions.

## Materials and methods

### Patients and clinical assessment

Ten patients were enrolled in this study. Informed consent was obtained from all participants. The study was approved by the ethics committee (Sud-Ouest et Outre-mer III; *n°2014-A00112-45*) and was in accordance with the tenets of the Helsinki Declaration. Inclusion criteria for VIM DBS were as follows: (1) suffering from severe and disabling ET; (2) 18–70 years old; (3) affiliated with the national social security system; (4) free of evolutive and untreated psychiatric disorder; (5) absence of depression (BDI < 10^24^); (6) cognitive functions preserved (score > 130 on Mattis scale^25^); (7) tremor predominating in upper limbs although possibly affecting lower limbs, head and voice. Exclusion criteria were as follows: (1) parkinsonian tremor; (2) pregnancy; (3) severe co-morbidity (cardiac, renal, or respiratory failure, evolutive cancer); (4) impaired cognitive functions (score < 130 on Mattis scale); (5) patients with severe atrophy or lesions on brain magnetic resonance imaging (MRI).

Tremor severity was assessed before surgery and 3, 12, 13 and 24 months after surgery both in “Off” and “On” stimulation, using the Fahn, Tolosa and Marin (FTM) tremor scale^26^. Patients were free of pharmacological treatment. For Off-DBS evaluations, FTM items 16 to 22 could not be assessed during the experimental session, so they were removed (feeding other than liquids, bringing liquids to mouth, hygiene, dressing, writing, and working). The Mattis and Beck scales were used to assess cognitive functions and patient’s mood before and 12 months after surgery. During the immediate post-operative period, some patients were partially or totally improved by a thalamotomy effect that prevented the testing of positive effects of DBS. These patients returned for a visit one month after surgery to optimize the stimulation settings in good conditions. For patients who exhibited a residual tremor, DBS was tuned with settings commonly used in the context of DBS for ET. On the other hand, side-effects were systematically documented in all the patients. One week after surgery, patients left hospital and thereafter returned for setting adjustments, if necessary.

### Surgical procedure

A Leksell frame (Elekta^©^) was positioned under local anaesthesia (lidocaine). 1.5 Tesla MRI (Siemens™ Achieva) was then performed using a T1 WI sequence (TR = 15 ms, TE = 7 ms, matrix 512 × 512, Flip Angle 30°, bandwidth 15 kHz, NEX = 1, slice thickness 1.5 mm). Coordinates of the VIM target were calculated in the (AC-PC) stereotactic space. These coordinates were: x = lateral wall of the third ventricle at the AC-PC level + 11.5 mm; y = 0.294 × length AC-PC in front of PC; z = − 1 mm (below AC-PC line). Details about the awake intra-operative procedure are the same as those published in the article by Engelhardt and colleagues^27^. Five days after electrode implantation, a second procedure was performed under general anaesthesia to place the stimulator (ACTIVA™ PC + S, Medtronic plc.) in the left sub-clavicular region and to connect it with the extensions and electrodes. Electrical connection between the stimulator and extensions was isolated using glue to increase the quality of the LFP signal.

### Post-operative localization of leads

Leads were located in the stereotactic space using the Bardinet-Yelnick atlas for reconstructions (SureTune III software, Medtronic Inc, Minneapolis)^28^. Patients underwent either a post-operative 3D T1 MRI on a SIEMENS ACHIEVA with the following parameters: 1.5 Tesla, SAR < 0.1 W/Kg, TR = 15 ms, TE = 7 ms, Matrix 512*512, flip angle 30°, bandwidth 15 kHz, slice thickness 1 mm; or a postoperative CT scan with 1 mm slice thickness which was co-registered with the preoperative MRI if a postoperative MRI was not available.

The DBS lead was traced on the postoperative imaging along the metallic artefact and automatically reconstructed by selecting the distal tip of the lead and a point chosen just proximal to the last active contact. Data was systematically confirmed by manual analysis of the coordinates given by the middle of each hypo-intensity shadow representing each contact. If two contacts were used simultaneously for stimulation, the middle of the two contacts was considered as the target. Coordinates were given in the conventional (AC-PC) space, with the MCP (mid-commissural point) as the origin. The location of DBS leads and active contacts according to surrounding anatomical structures (i.e., Vim and other thalamic nuclei) was analysed and SureTune III was used for image processing. Active contacts of the patients included in this study were plotted in 3D, axial, sagittal and coronal views in the stereotactic referential, and mean coordinates were calculated using Matlab^©^ (MathWorks™).

### Acquisition of local field potentials

Three, 12, 13 and 24 months after surgery, LFPs recordings started after impedance have been checked. Post-operative neurophysiological assessment was performed under standardized clinical situations: 30 s at rest in a standing position with the arms hanging on either side of the body, and during clinical conditions favoring the emergence of tremor: 30 s in a standing position with the arms outstretched in front of the body, 30 s in a standing position when holding the arms abducted, up in the air with elbows flexed and the fingers of both hands pointing towards each other, 30 s in a seated position by pouring water from one glass to another, 30 s in a standing position during the finger-to-nose test with the right arm and then 30 s with the left arm. Except for the finger-to-nose test where a mean of ten round trips were realized with each hand, all other clinical tests were repeated three times, interspersed with rest periods of 30 s to 1 min to avoid patient fatigue. Thus, two main clinical conditions were established: the rest condition and clinical conditions inducing tremor (posture maintenance and action). The total duration of the experimental protocol was 3h at each recording session, i.e. 3, 12, 13 and 24 months after surgery.

### LFP recordings

The ACTIVA™ PC + S from Medtronic was used for LFP recordings. The ATIVA™ PC + S system is a multiprogrammable device that both delivers electrical stimulation and records bioelectric data. Sensing and the recording of bioelectric data are controlled by a separate sensing clinician programmer. The latter consists of a tablet computer and a telemetry head that connects to the tablet via a USB port. The sensing programmer allows the operator to control amplifier settings on the ACTIVA™ PC + S including gain, low pass filters, high pass filters, sampling frequency, and, in frequency domain, the frequency band^29^. Data is stored in the implantable neurostimulator (INS) as bipolar bioelectric voltage recorded in time domain or frequency domain. Recorded bioelectric data is extracted from the INS by telemetry using the sensing programmer. Data can then be transported to another computer for off-line analysis.

### “Off” stimulation recordings (Off-DBS)

“Off” stimulation recordings sessions were performed 3 and 12 months after surgery. Additional short “Off” stimulation recordings sessions were performed 13 and 24 months after surgery, just before the “On” stimulation recording session. The left lead contacts were numbered from E0 to E3 and those of the right lead from E8 to E11. Twenty minutes after stimulation was turned Off, LFP recordings in the time domain-422Hz were performed at the same time for both right and left leads as follows: firstly, using bipolar montage between E0-E1 and E8-E9, secondly between E1-E2 and E9-E10 and thirdly between E2-E3 and E10-E11. For each bipolar montage and in each clinical condition described above, 30 s of LFP acquisition were repeated in triplicate.

### “On” stimulation recordings (On-DBS)

One contact was used for stimulation and the two surrounding contacts were used for bipolar LFP recordings^29^. For example, if E1 was used for stimulation, LFP acquisition was performed between contacts E0 and E2. When possible, the contact used of chronic stimulation was selected for this condition. If this was not possible (e.g. contact E0 used for chronic stimulation), the contact located just above or below was selected (E1 in our example). Thus, in this situation, the population of neurons recorded Off- and On-DBS was admittedly not completely the same. To measure the neural signature of interest during VIM stimulation, power was extracted in the frequency domain between 0.5 to 45 Hz*.* Power in this window was averaged over 30 s. Stimulation was delivered at 130 Hz with a pulse duration comprised between 60 and 90µs according to the settings used for chronic stimulation. On-DBS recordings were performed in all patients 13 and 24 months after surgery. However, we were not systematically able to configure appropriately the setting of recordings (gain and filters) during stimulation. Consequently, large stimulation artefacts were present and hampered the analysis of the signal in some patients, interfering with LFPs analysis in 2 patients (P4, P10) 13 months after surgery and in 5 patients (P2, P4, P6, P7, P9) 24 months after surgery.

### Signal processing

All the pre-processing steps were done using EEGLAB functions^30^ and Matlab scripts (MATLAB_R2017; *version 9.2.0.556344* Natick, Massachusetts: The MathWorks Inc.). Raw LFP recordings in xml and txt format were imported into Matlab, then synchronized using pulses at the onset of recording. The accelerometer sampling rate was changed to be identical to that of the LFP recordings. After this step, we obtained a 30-s single file per experimental condition. The first 7 s of each recording were discarded because they contained only the synchronization pulses. On some recordings, the ACTIVA™ PC + S captured cardiac activity that masked brain signals. The amplitude of this EKG signal is a few hundred mV, and its frequency spectrum is in the 0.5–45 Hz band, so it cannot be filtered directly. We first checked whether there were any EEG artifacts from EKG (R wave, QRS-width, and ST interval) activity. Once these were identified, the average QRS complex and the T-wave were calculated. The average was fitted to each single artifact identified separately and subtracted from the original signal. The LFP channels were filtered from 2 to 50Hz (EEGLAB pop_eegfilnew that filter data using Hamming windowed sinc FIR filter) and a DC removal was applied before normalization by removing the average from each channel. Post-surgery anatomical reconstructions enabled us to identify the channel corresponding to the different anatomical structures. By locating the four contacts of each lead, we could categorize the bipolar montages used for LFP recordings: inside target (IT) when at least one of the two contacts used for the bipolar montage was localized within the VIM; therapeutic contact (TC) when at least one of the two contacts used for the bipolar montage was used for chronic stimulation within the VIM; and outside target (OT) when the two contacts used for the bipolar montage were located outside the VIM. All the cleaned and identified LFP recordings were added to a study structure in EEGLAB. Spectral analysis of all files was performed in EEGLAB study mode using FFT between 0.5Hz and 50Hz. A peak in the frequency band of interest was considered as significant when PSD amplitude was > 0.2 µV^2^/Hz.

### Accelerometry

Tremor was assessed using a 3-Axis accelerometer (Phidgets 1059, Calgary, Canada) accelerometer 3, 12, 13 and 24 months after surgery. It was positioned on the index of the hand where the tremor amplitude was the highest. Data was continuously recorded using the Oyapock toolbox (Matlab, MathWorks, Natick, USA) at 50Hz.

### Posturography

Balance was evaluated because ataxia is a symptom commonly encountered in ET and sometimes increased by VIM DBS. Then, posturography was performed before surgery and 3, 12, 13, 24 months after surgery when stimulation was “On”. Subjects remained erect on a multi-component force platform (AMTI, USA, 50 Hz) to record the position of their center of pressure (CoP) when their eyes were open. For each patient, the position of the feet was drawn at the first recording and was identical for all the subsequent tests. Postural parameters were based on the suggestions of Prieto et al.^31^. The area of the 95% confidence ellipse and the CoP traveled distance in 2D were calculated with Awara Matlab toolbox.

### LFP synchronization with accelerometry and posturometry

LFP recordings of 30 s duration and in the time domain (422Hz) were started manually with the Medtronic remote control. Using Oyapock Toolbox (Matlab), AMTI forceplate and Phidget accelerometer were recorded simultaneously. To synchronize these behavioral data with LFP recordings, 5 short, small electrical pulses (3mA, 2ms, 1Hz) were applied to the patient’s skin to generate electrical artifacts on thalamic recordings. The anode was placed near the implantable pulse generator (IPG-Medtronic) and the cathode over the extension that passed into the mastoid region. We used “2 × 2 cm” electrodes (Dura-Stick 42190). Stimulation was provided by a constant current generator (Digitimer DS5, Letchworth Garden, United Kingdom), and a copy was recorded in Oyapock (Matlab) on an A/D card (National Instrument USB-6363). For off-line analysis, this system was used to synchronize behavioral data with LFP recordings marked by the electrical artifact.

### Statistical analysis

A descriptive analysis of the FTM scores was made before and 3 and 12 months after surgery. FTM data obtained before and after surgery were compared with the Wilcoxon rank test in which p < 0.05. For accelerometry, amplitude of the power spectrum density (PSD) was compared before and after surgery in the different clinical conditions using a Student t-test in which p < 0.05 because there was high amount of data with a normal distribution (EEGLAB statistic toolbox). For the sake of clarity, we separated the rest condition from clinical conditions inducing tremor: position arm outstretched in front of the body was pooled with position of arms abducted, up in the air with elbows flexed and the fingers of both hands pointing towards each other (called “postural condition”), and the pouring of water from one glass to another was pooled with the finger-to-nose test (called “action condition”). Thus, clinical conditions inducing tremor were called the ‘posture/action’ condition. We calculated a mean PSD from the two or three LFP recorded in each clinical condition and for each bipolar montage. Then, we compared the LFP-PSD amplitudes acquired (frequency resolution of 1 Hz) in the two clinical conditions (rest, posture/action) using a Student t-test in which p < 0.05 (EEGLAB statistic toolbox). All statistical analyses on spectra were carried out in EEGLAB study mode with a t-test depending on the comparison with a p value set to p < 0.05 and after Bonferroni correction. For further analysis, we separated the data into different frequency bands = delta (0.5-3Hz), theta (4-7Hz), alpha (8–12 Hz) and beta (low-beta 13–20 Hz and high-beta 20–35 Hz). We performed the equivalent of an ANOVA analysis for small samples of data (Kruskal–Wallis test) taking LFP-PSD amplitude into account for the different frequency bands, the site of LFP recordings (IT vs TC), the clinical condition of recordings (rest vs posture/action) and the recording endpoint time (M3, M12 and M24).

## Results

### Sites of recordings and stimulation

**Figure 1:**
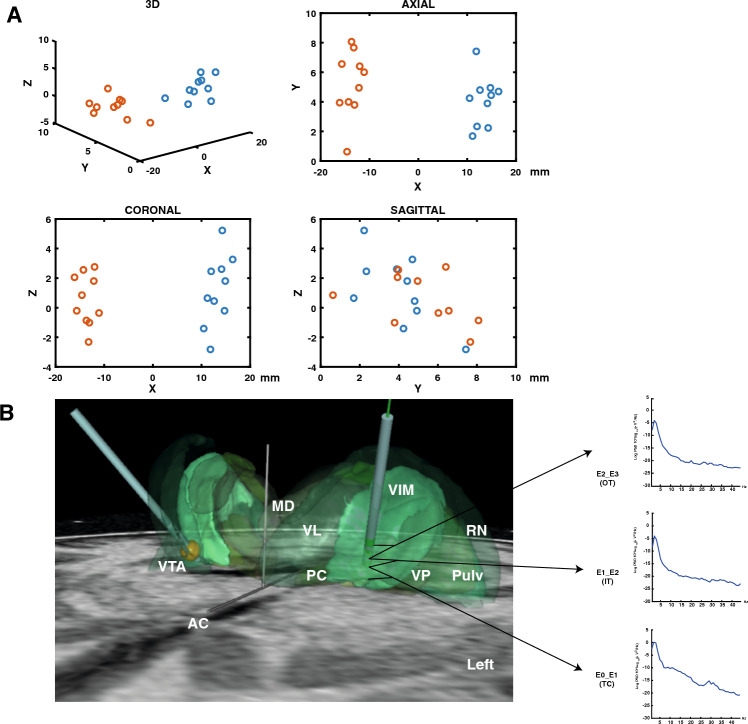
Contacts used for deep brain stimulation. (**A**) Stereotactic coordinates (x, y, z) of contacts used for deep brain stimulation (DBS). Red circles correspond to contacts used for chronic stimulation on the left side. Blue circles correspond to contacts used for chronic stimulation on the right side. These coordinates are obtained in relation to the mid-commissural point (the middle of the AC-PC line). For the z axis, negative values correspond to coordinates located below the AC-PC line (AC = anterior commissure; PC = posterior commissure). (**B**) Example of lead placement within the ventral intermediate nucleus (VIM) for P1. Examples of local field potentials (LFP) recorded during posture between the two adjacent contacts from bottom to top of the left lead (E0E1, E1E2 and E2E3). In this patient, E0E1 corresponds to a bipolar montage of the therapeutic contact (TC) type because E0 was used for chronic VIM stimulation. The bipolar montage E1E2 was considered as intra-target (IT) because E1 and E2 were located inside the VIM but not used for chronic DBS. The bipolar montage E2E3 is of the outside target (OT) type because E3 was located outside the VIM. In this example, high-beta oscillations (28 Hz) were observed only from the TC bipolar montage (between E0_E1). The right lead shows an example of the volume of tissue activated (VTA) using 1.7 Volt, 60 µs of pulse duration and 130 Hz through the deepest contact (E8). *AC* anterior commissure, *PC* posterior commissure, *MD* medio-dorsal nucleus, *VL* ventral lateral nucleus, *VIM* ventral intermediate nucleus, *VP* ventral posterior nucleus, *RN* reticular nucleus, *Pulv* pulvinar nucleus.

The best tremor improvement was generally obtained using stimulation contacts located within the VIM (8/10 patients for the left and 9/10 patients for the right thalamus). For two patients (P7 and P9), contacts were located just below and lateral to the left VIM (Fig. 1A). For one patient (P4), one contact was located below the VIM near the right subthalamic region. The mean values of x, y and z coordinates related to the mid-commissural point were x = 13.6 ± 1.6, y = 5.2 ± 2.2, z = 0.53 ± 1.7 mm on the left side and x = 13.3 ± 1.9, y = 4.1 ± 1.7 and z = 1.2 ± 2.4 mm on the right side. Using the SureTune III software, we localized the four contacts of each lead. Figure 1B illustrates the location of the right and left leads for P1 and an example of LFP recorded from the three bipolar montages of the left lead (IT, OT, TC), three months after surgery during posture maintenance.

### Effectiveness of VIM-DBS and side-effects

The epidemiological characteristics of patients and DBS settings are reported in Table 1. Twelve patients with essential tremor were enrolled in the present study. Two were excluded because of device infection which occurred within the 3 months following pulse generator implantation. The left lead of one patient (P10) moved 15 months after surgery and was then replaced under general anaesthesia. The patient’s clinical status returned to that after the initial procedure. Drugs used for tremor before surgery were beta blockers (propranolol 80-160mg/d) only or in association with primidone (125–1250mg/d). Patient P1 was treated with gabapentin (1500mg/d). Pharmacological treatments were stopped during the 3-month postoperative period in all patients.

**Table 1:** Epidemiological characteristics of patients and DBS settings.

	DD	FTM		Mattis		BDI		Left lead				Right lead			
		PreOp	M12	PreOp	M12	PreOp	M12	Contact	V	PW	Fq	Contact	V	PW	Fq
P1	30	32	0	142	141	17	9	0–	1.5	60	130	8–	1.7	60	130
P2	43	56	36	139	139	5	3	1–	2.8	120	130	9–	2.7	90	130
P3	7	46	14	138	138	ND	2	0–	1.8	60	130	8–	1.3	60	130
P4	44	31	6	141	141	0	2	0–	2.2	60	130	8–	2	60	130
P5	3	34	4	137	132	12	6	2–	1.1	90	130	10–	1.7	60	130
P6	12	50	19	143	142	8	10	1–2–	2.8	90	130	8–	1.3	60	130
P7	44	39	14	134	140	12	19	0–	1	60	140	8–	1.4	60	140
P8	19	44	14	136	136	7	0	0–	1.7	60	130	8–	3.4	60	130
P9	26	32	8	140	142	ND	8	0–	2	60	130	8–	2.3	90	130
P10	12	62	30	139	139	10	5	2–	2.7	90	130	9–	2.7	60	130
M	24.0	42.6	14.5	138.9	139.0	8.9	6.4	/	2.0	75.0	131.0	/	2.1	66	131
SD	15.8	10.9	11.3	2.8	3.1	5.1	5.5	/	0.7	21.2	3.2	/	0.7	12.6	3.2

*DD* disease duration (years), *FTM* Fahn, Tolosa and Marin Tremor score before (PreOp) and 12 months after surgery (M12), *Mattis* Mattis scale, *BDI* Beck Depression Inventory, *Contact* contacts used for chronic deep brain stimulation (DBS), *V* voltage used for chronic DBS, *PW* pulse width used for chronic DBS, *Fq* frequency of DBS, *M* mean, *SD* standard deviation, *ND* no data available.

**Figure 2:**
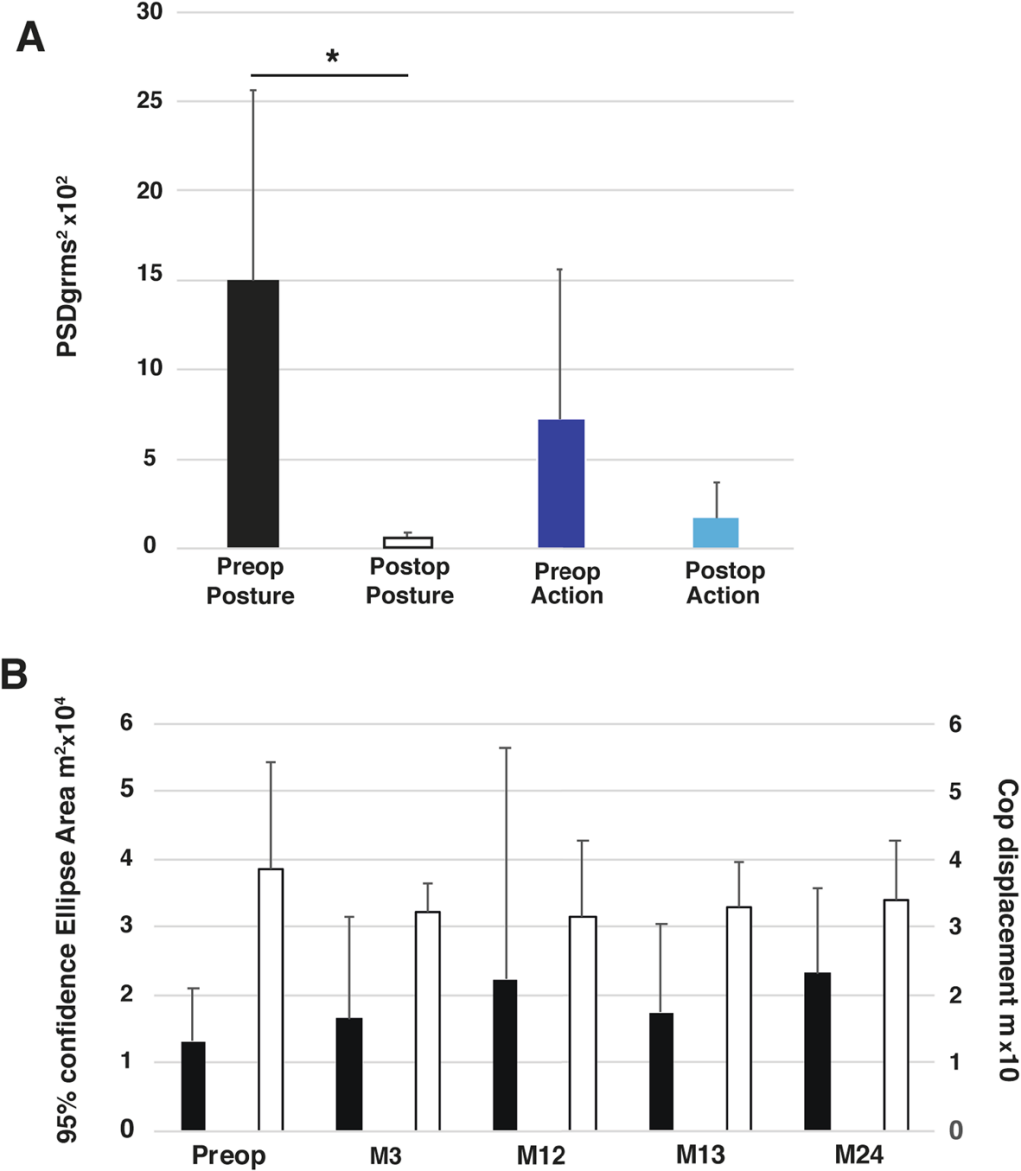
VIM-DBS clinical effectiveness and its impact on balance. (**A**) Accelerometry. Comparison of power spectrum density (PSD**g**_**rms**_^**2**^) for posture and action, before and 12 months after surgery. There was a significant decrease in postural tremor after surgery (15 × 10^–2^ ± 10 × 10^–2^ vs 0.5 × 10^–2^ ± 0.4 × 10^–2^ PSDg_rms_^2^ before and 12 months after surgery respectively; p = 0.0001). (**B**) Posturography. Black bars represent 95% confidence ellipse area (m^2^) and white bars correspond to Centre of pressure (Cop) length displacement (m). VIM-DBS did not induce any significant change of these parameters, whatever the time of post-operative assessment.

The effectiveness of DBS was assessed using the Fahn, Tolosa and Marin tremor scale (FTM). Comparison of FTM total score before and 12 months after surgery revealed an improvement in tremor of 66% (42.6 ± 10.9 and 14.5 ± 11.3; p = 0.005; Table 1). Regarding upper limb FTM scores during posture and action, the improvement was 76.2% and 63.6%, respectively. Analysis of accelerometer data during posture [power spectrum density (PSDg_rms_^2^)], revealed a mean decrease in tremor amplitude of 96.1% (15 × 10^−2^ ± 10 × 10^−2^ vs 0.5 × 10^−2^ ± 0.4 × 10^−2^ before and 12 months after surgery respectively; p = 0.0001; Fig. 2A). During movement execution (action), there was a non-significant decrease in tremor amplitude of 80.8% (7 × 10^−2^ ± 8 × 10^−2^ vs. 1 × 10^−2^ ± 1 × 10^–2^ before and 12 months after surgery respectively; p = 0.13; Fig. 2A). No depression nor cognitive impairment were observed (BDI scores 8.9 ± 5.1 before and 6.4 ± 5.5 twelve months after surgery; p = 0.29; Mattis total scores 139.2 ± 2.6 before and 139.0 ± 3.1 twelve months after surgery; p = 0.89; Table 1). Side-effects related to stimulation were occasionally observed. One patient complained of moderate dysarthria, and two patients exhibited slight ataxia 3 and 12 months after surgery but did not need physiotherapy. VIM-DBS did not induce any significant change in balance quality, as suggested by the stability of the center of pressure (CoP) displacement (Fig. 2B). The area of 95% confidence ellipse was not influenced by VIM-DBS, whatever the time of post-operative assessment (Fig. 2B).

### Local field potential recordings

**Figure 3:**
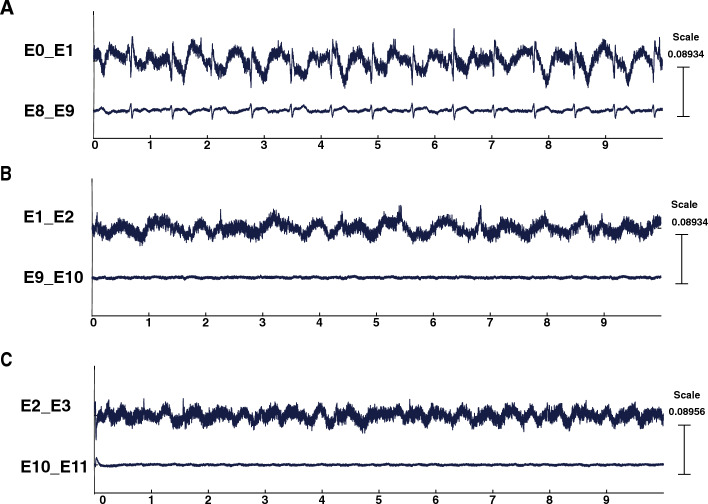
EKG artifacts. Example of raw data recorded with bipolar montages successively from E0_E1/E8_E9 (**A**), E1_E2/E9_E10 (**B**) and E2_E3/E10_E11 (**C**). EKG artifacts are commonly observed through the deeper montages between E0_E1 on left side and between E8_E9 on right side. Amplitude of EKG signal was a few hundred mV, and its frequency spectrum was within low delta band (1–2 Hz).

**Figure 4:**
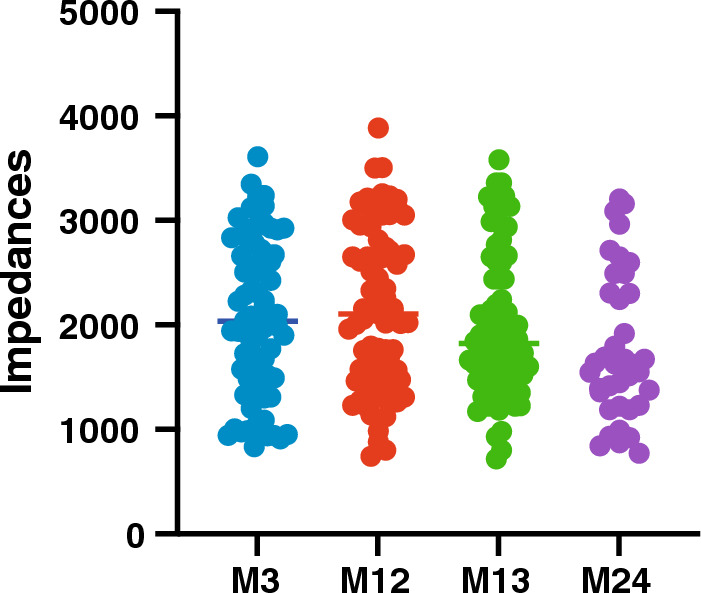
Time evolution of impedances. Impedances were measured just before LFP recording sessions. Each dot corresponds to the impedance value of one tested contact from the right and left leads pooled and at different timepoints (from M3 to M24). Impedances > 40 000 Ohms not reported on this graph.

LFPs were recorded Off stimulation 3, 12 and 13 months after surgery in all patients and in 6 of them 24 months after surgery (P1, P2, P3, P5, P8, P10). LFPs were recorded On stimulation 13 months after surgery in all patients except P4 and P10 and in 5 patients 24 months after surgery (P2, P4, P6, P7, P9). EKG artifacts were frequently observed through the deeper montages (between E0_E1 on the left side and between E8_E9 on the right) in most patients (Fig. 3). Finally, one or two contacts were not used for LFP recordings in two patients because of high impedances > 40 000 Ohms (contact E10 of the right lead for P4 and contacts E3 and E10 of the left and right leads, respectively for P8). For the remaining patients, impedances were not significantly different over time for each tested contact from the two leads (P = 0.067 Kruskal–Wallis test; Fig. 4).

### Characteristics and time course of VIM LFPs when stimulation was Off

For the sake of clarity, we reported thalamic oscillations recorded from contacts located only within the VIM (contacts used or not used for chronic DBS: IT + TC). Because posture and action are clinical conditions involving both arms and because the results were similar between recordings performed on both sides, we decided to pool the data from both hemispheres. When stimulation was Off, oscillations in different frequency bands of interest were recorded in the VIM of our 10 patients at rest, in the absence of tremor or during clinical conditions inducing tremor (posture/action). However, differences depending on the assessment time and the frequency band could exist. Two kinds of results can be distinguished: 1/ oscillations that were absent at rest but appeared during posture and action; 2/ oscillations that were present at rest and persisted during posture and action.

**Figure 5:**
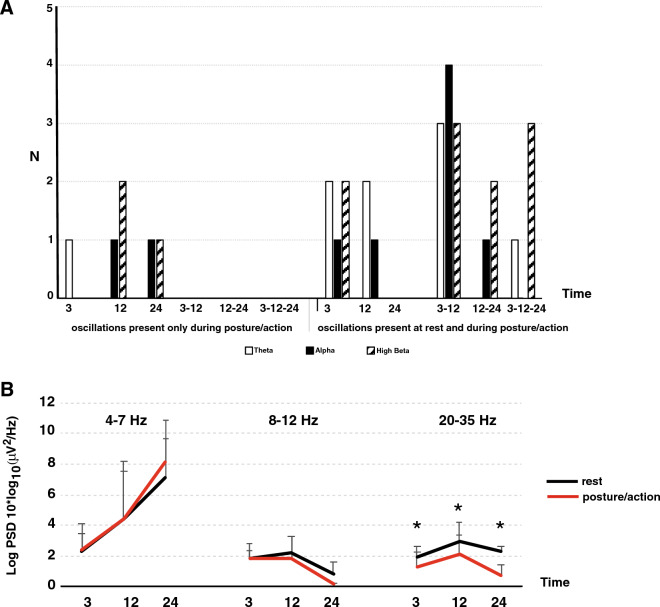
Time evolution of LFPs. (**A**) Number of patients (N) exhibiting theta, alpha and/or high-beta thalamic oscillations within the VIM (right and left side pooled) 3, 12 and 24 months after surgery. Left part corresponds to oscillations appearing only during posture/action. Right part corresponds to oscillations present at rest as well as during posture/action. (**B**) Time course of power spectral density (PSD) amplitude over time from 3 to 24 months in the different frequency bands of interest, at rest (black line) and during posture/action (red line). Time in months. *p ≤ 0.01.

**Figure 6:**
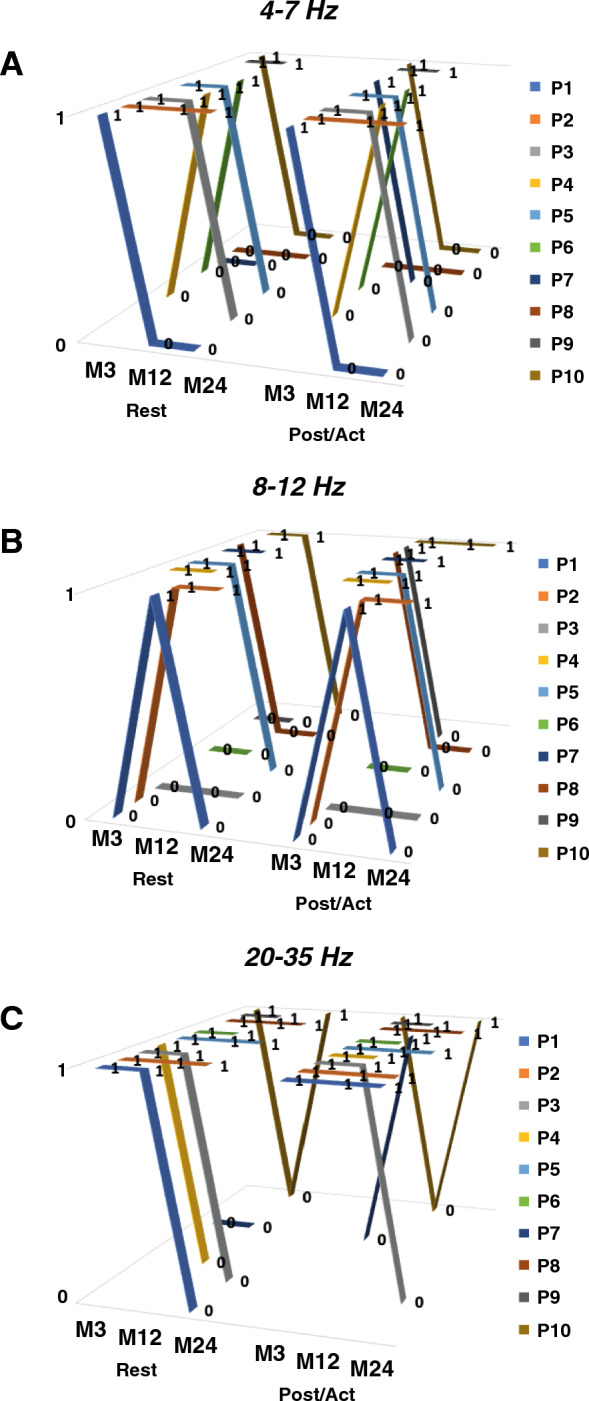
Evolution of thalamic oscillations over time for each patient. Presence (1) or absence (0) of VIM LFPs in the different frequency bands (theta: 4-7Hz; alpha: 8-12Hz; high-beta 20–35 Hz) and their time course (3, 12 and 24 months after surgery) at rest and during posture/action (Post/Act), in patients P1 to P10.

A few patients had thalamic oscillations appearing only during posture/action, whatever the frequency range (Fig. 5A, left part). For these patients, thalamic oscillations were recorded only at one time during follow-up (3, 12 or 24 months after surgery in P1, P4, P7 and P10). On the other hand, the presence of theta, alpha or high-beta oscillations was observed at rest as well as during posture/action either at one time during follow-up (3 or 12 months after surgery in P1, P4, P6, P8 and P10) or on a recurring basis, two or three times during follow-up (3 and 12 months in P3, P4, P5, P6, P7, P9 and P10; 12 and 24 months in P1, P2 and P10; and 3, 12 and 24 months after surgery in P2, P5 and P8; Fig. 5A, right part). Evolution of thalamic LFP in each frequency band over time for each patient is available in Fig. 6.

**Figure 7:**
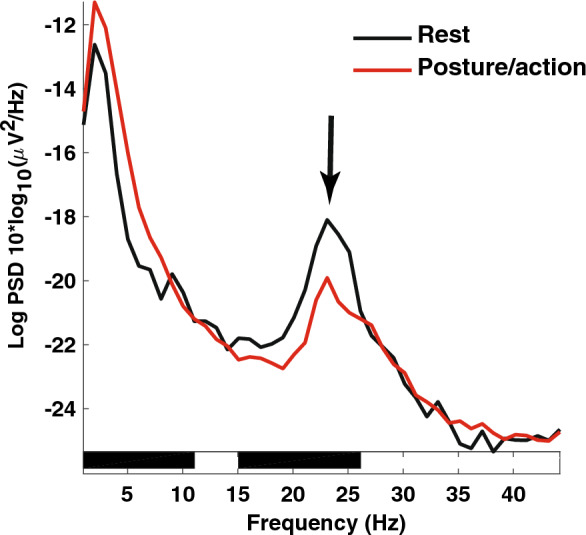
Effects of posture maintenance and movement on LFP PSD amplitude. LFP recorded in P6 from a montage including a contact used for chronic stimulation (TC) on the right side. High-beta-LFP (arrow, 23Hz) PSD amplitude decreased during posture/action (red line) comparing to the rest condition (black line). Horizontal black bars on the *x* axis correspond to a statistical difference with p = 0.0001. *PSD* power spectrum density. LFP recorded 3 and 12 months after surgery were pooled for this analysis.

Regarding oscillations that were present at rest and persisted during posture/action, we analysed their PSD amplitude to track significant modulations related to clinical conditions. There was no significant difference in PSD amplitude for the theta and alpha bands between the rest condition and posture/action 3, 12 or 24 months after surgery (p = 0.64 for theta and p = 0.47 for alpha band, Kruskal–Wallis test; Fig. 5B). On the other hand and compared to the rest condition, there was a significant decrease in PSD amplitude in the high-beta band during posture/action 3, 12 and 24 months after surgery (p < 0.001, Kruskal–Wallis test; 3 months after surgery 1.91 ± 0.89 at rest vs. 1.27 ± 1.37 µV^2^/Hz during posture/action in 8/10 patients; p = 0.010; 12 months after surgery 2.92 ± 1.75 at rest vs. 2.12 ± 1.78 µV^2^/Hz during posture/action in 7/10 patients; p = 0.014; at 24 months 2.32 ± 0.35 vs 0.75 ± 0.78 µV^2^/Hz rest and posture/action respectively in 4/6 patients; p = 0.017; Mann–Whitney test; Fig. 5B). High-beta oscillations occurred at least twice during the 24 months of follow-up in 8 of the 10 patients. Figure 7 shows LFPs recorded in the right VIM from a montage including a contact used for chronic stimulation (TC for therapeutic contact) in P6 (we pooled the data obtained 3 and 12 months after surgery). In this example, the PSD amplitude of a peak in the high-beta band (23Hz; arrow) significantly decreased during posture/action in comparison to the rest condition.

There was no correlation between the amplitude decrease in PSD (between rest and posture/action) in the high-beta band recorded at M3, M12 and M24, and the amplitude of FTM improvement (difference between pre-and M3, M12 or M24 post-op; p > 0.05 Spearman tests).

### Effects of DBS on VIM LFPs

**Figure 8:**
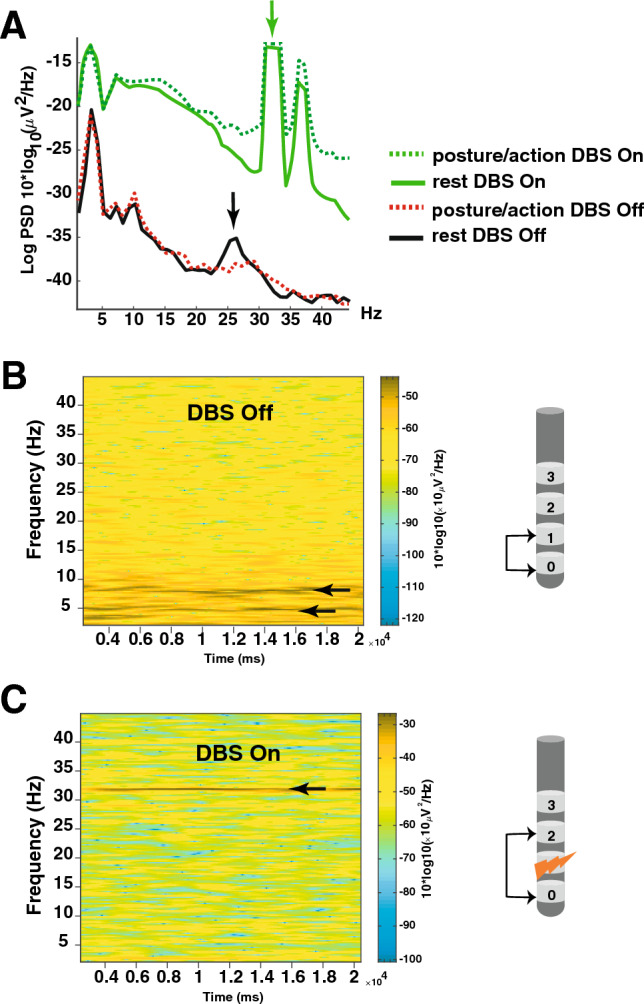
Effects of VIM-DBS on LFP. (**A**) High-beta LFPs (black arrow) recorded Off stimulation in P1 on the left side at rest (black solid line) and during posture/action (red dotted line). LFPs recorded On stimulation at rest (green solid line) and during posture/action (green dotted lines). Green arrow: stimulation artifact. (**B**) Time–frequency representation of the spectrum from 2 to 45 Hz. Note theta-LFPs (5Hz, lower horizontal arrow) and their harmonics (10Hz, upper horizontal arrow) recorded Off stimulation from the left lead of P2 (between the contacts 0 and 1) during posture maintenance 13 months after surgery. (**C**) Time–frequency of LFP recordings in the same clinical condition but when the stimulation was turned On, in the absence of tremor (stimulation delivered in the left VIM through contact 1 and recordings performed between contacts 0 and 2). DBS settings (2.8V, 120µs, 130Hz). Sub-harmonic of DBS artifact (black horizontal arrow). *PSD* Power spectrum density.

The effects of DBS on VIM LFPs were studied in patients who previously exhibited thalamic oscillations at rest and/or during tremor when stimulation was Off (7/10 patients). However, LFPs in the theta or alpha bands were not systematically observed in these patients so the VIM-DBS findings at these frequencies cannot be generalized. Twenty-four months after surgery, only 5 patients could be tested for technical reasons (gain and filters setting difficulties) and only their beta-LFPs. In addition, the presence of significant stimulation artifacts made the analysis of raw data and the interpretation of LFPs difficult. However, during VIM stimulation, and whatever the clinical condition, thalamic oscillations disappeared in the theta, alpha and high-beta bands. Figure 8 illustrates high-beta-LFPs (black arrow) recorded Off stimulation in P1 on the left side at rest (black line) and decreased by posture/action (red dotted line; Fig. 8A). When stimulation was switched On, high-beta-LFPs were suppressed at rest as well as during posture/action (green solid and dotted lines respectively; Fig. 8A). Figure 8B,C shows a time–frequency representation to illustrate the effect of VIM-DBS on theta-LFPs (5Hz) and their harmonics (10Hz) recorded from the left lead in P2 during posture maintenance 13 months after surgery. This activity abated as tremor improved.

## Discussion

LFPs were recorded in long-term conditions in the thalamus of 10 ET patients, thanks to an implanted system allowing both LFP recordings and chronic stimulation (ACTIVA™ PC + S). Independently of the frequency band, only a few patients (4/10 patients) experienced thalamic oscillations during clinical conditions inducing tremor. In addition, none of them exhibited this phenomenon on a recurring basis over time. On the other hand, regarding the entire frequency range, all patients exhibited persistent thalamic oscillations at rest and during posture/action at least twice during the follow-up. In these patients, high-beta activity was the only thalamic frequency that was significantly decreased by clinical conditions inducing tremor 3, 12 or 24 months after surgery, suggesting that it could be a suitable parameter for closed-loop therapy in ET.

### Fate of thalamic oscillations over time when stimulation was Off

We recorded thalamic oscillations that were absent at rest but appeared during posture and action, i.e. movements inducing tremor in daily life. However, this phenomenon was observed in only 4 of the 10 patients, whatever the frequency band, and was not sustainable over time. Theta/alpha-LFP in the frequency range of tremor was previously reported in the VIM of patients with ET using externalized electrodes or macroelectrodes during surgery^19^. However, these thalamic oscillations in the range of tremor or in harmonic frequencies were recorded in only about a third of patients^32–34^. Similarly, single-unit recordings revealed the existence of tremor cells in 35–56% of neurons tested during postural tremor^35,36^. Thus, the detection of thalamic oscillations occurring only during tremor would not appear to be generalizable in closed loop therapy in the future, since the oscillations were not systematically observed in all the patients. Moreover, when they were observed, they were not always reproducible over time.

Secondly, we recorded thalamic oscillations at rest in the absence of tremor, but which persisted during posture and action. Those in the high-beta band were the only ones that were significantly decreased by movements inducing tremor, so they are potentially suitable for closed-loop therapy. We already demonstrated that the PSD amplitude of beta-LFPs was significantly decreased by movements inducing tremor 3, 12 or 24 months after surgery. Decreases in averaged beta power have been observed over the sensorimotor cortex (SMC) both intracranially and on the scalp^37–40^, the STN^41^ and the ventral intermediate (VIM) nucleus of the thalamus^42^ during voluntary movement execution, as well as a reduction in beta burst rates in the SMC and STN of PD patients^14^. Changes in movement-related beta activity have been previously reported in the thalamus of ET patients during self-paced movements or when performing a Go/noGo task shortly after surgery^18,43^. This data suggests that a physiological event-related desynchronization caused by postural or action movements could explain the reduced high-beta activity observed in our study. Interestingly, beta-LFPs have also been recorded in the subthalamic nucleus (STN) in akinetic patients suffering from PD, suggesting that beta activity is clearly linked to the scarcity of movement^44^. This hypothesis is strengthened by the decrease in beta-LFP amplitude after levodopa intake^45–48^ or STN-DBS^49–52^ in parallel with improvement in akinesia. Thus, regardless of the underlying neurological disease, movement-related beta-LFPs could potentially be used as biomarkers of movement in closed-loop therapy in the future. Opri and collaborators tested closed-loop stimulation for VIM DBS (CL-DBS) in three patients with ET over a period of 6 months after surgery. They delivered VIM stimulation based on upper limb motor activity detected with a cortical electrode and demonstrated that this system was as effective as open-loop stimulation^23^. In two patients with ET, Ferleger and collaborators collected VIM gamma-LFPs and cortical beta-LFPs in order to test a linear classifier for closed-loop therapy^53^. They concluded that adaptive DBS (aDBS) was 33.2% more effective than chronic DBS (cDBS). However, they also mentioned that classifier longevity, defined as the number of weeks a given classifier remains clinically effective, will require longitudinal studies. Recently, He and colleagues addressed the question of closed-loop therapy based on thalamic LFP in ET^54^. Their study was based on a binary classifier machine learning process to characterize VIM LFP activity^19^. Using an offline analysis, they detected a decrease in LFP activities related to voluntary movements (13-23Hz beta) and an increase in LFP activities related to postural tremor (4-7Hz theta). They tested whether their models could detect movement and tremor in real time in order to switch DBS on. They concluded that both voluntary movements and postural tremor can be detected online with a sensitivity around 80%^54^. Applying their models shortly after surgery in 8 ET patients with externalized leads, they recently performed successful closed-loop therapy based on tremor-provoking movement detection with a similar amount of tremor suppression as with continuous stimulation^55^.

Nevertheless, all the above-mentioned data was obtained from recordings performed just after surgery or 6 months after it. Thus, it was crucial to verify whether beta-LFPs were detectable over time to be sure that closed-loop therapy can be administered in the long term based on this specific activity. Here, we demonstrate that 8/10 patients 3 months after surgery, 7/10 patients 12 months after surgery and 4/6 patients 24 months after surgery, exhibited a decrease in high-beta PSD amplitude during movements inducing tremor. All of them exhibited this phenomenon at least twice during the 24 months of follow-up. Although our sample of patients is small, this result suggests that the detection of a decrease in high-beta PSD amplitude induced by voluntary movement over a long period of time could be a relevant strategy for closed-loop therapy in patients with ET.

### What happens to thalamic oscillations during VIM-DBS?

VIM-DBS decreased the amplitude of theta, alpha and beta LFPs at the same time as tremor improved, thus raising questions about a potential link between these oscillations and their involvement in the pathophysiology of tremor. In a recent intraoperative study, Milosevic and collaborators observed inhibition of the thalamic firing rate and a decrease in LFPs in the frequency range of tremor during VIM stimulation in parallel with an improvement in tremor^36^. We did not find any correlation between the amplitude decrease in PSD (between rest and posture/action) in the high-beta band recorded at M3, M12 and M24, and the improvement in FTM, i.e. difference between pre-and M3, M12 or M24 post-operative values. This could be due to the fact that the recordings were performed in different clinical conditions and concerned a frequency different from the frequency range of tremor. The amplitude decrease in PSD in the high-beta band was assessed in the VIM-DBS Off condition, whereas the improvement in FTM was assessed in the VIM-DBS On condition.

## Limitations

This study has some limitations. Although our patient sample is small, it is large enough to verify the main objective, i.e. whether the electrophysiological characteristics of the thalamic oscillations of each patient remain the same and are reproducible over a long follow-up. On the other hand, we chose to characterize VIM-LFPs in separate clinical states independently of each other (at rest, during posture/action). For this reason, we did not obtain any data about the dynamic of LFP modulations when the patients moved from rest to action or from rest to posture inducing tremor. It would be interesting to investigate these status changes to verify whether the onset of voluntary movements and postural tremor can be predicted by specific changes in LFP characteristics and if so, if DBS could be switched on before the onset of tremor. Owing to a stimulus artifact, we had difficulties in studying the effects of DBS on thalamic oscillations and in all the frequency bands. Thus, the findings on the effects of VIM-DBS on LFPs should be taken with caution. Similarly, caution is required regarding the feasibility of recording VIM-LFPs when stimulation is On. This problem will probably be overcome soon thanks to the new generation of stimulators (PERCEPT; Medtronic). This issue is crucial because we must be able to monitor thalamic LFPs during DBS in order to switch DBS off when the patient returns to the resting state, and consequently no longer exhibits tremor. Buijink and collaborators recently demonstrated the feasibility of thalamic LFPs recordings using the electrodes and pulse generator of an implanted DBS system (PERCEPT from Medtronic)^56^. An additional limitation concerns the methods used to record LFP without or during stimulation. Indeed, when stimulation was Off, LFP recordings were performed with different montages than those used in the On-stimulation condition. This raises the question of the possibility of recording the same population of neurons in these two conditions. The first goal of this study was to characterize neuronal activity without stimulation in different clinical conditions over time and at each level of the leads (short-distance bipolar montages). This strategy allowed recordings between closely spaced contacts to be sure that LFPs were generated by neurons located at least around the contact selected for chronic stimulation and that gave the best clinical result. When stimulation was On, we had to record on both sides of the contact used for chronic stimulation (long-distance bipolar montages) as recommended by Medtronic to reduce the stimulation artefact. We hope this problem is solved with the new generations of PERCEPT, which will make it possible to record with the same contacts in Off and On-stimulation. Finally, the frequent occurrence of EKG artifacts may prevent from automatic online thalamic LFP analysis for closed-loop application.

## Conclusion

Although the amplitude of thalamic beta-LFPs is known to be decreased by voluntary movement or posture maintenance in patients suffering from ET, it was relevant to demonstrate that beta-LFPs persist over time when stimulation was Off, a phenomenon which is a prerequisite for its use in closed-loop therapy. However, the latter was not systematically observed in all patients. Consequently, the current findings need to be replicated in a larger number of patients to verify their interpersonal variability. To this end, new implanted DBS systems allowing the chronic sensing of thalamic LFPs should provide new data on the feasibility of such routine recordings in an ecological way and over a long period of time. Such technological evolutions should pave the way for personalized closed-loop therapy based on thalamic activity.

## Data Availability

The datasets used and/or analyzed during the current study are available from the corresponding author on reasonable request.
